# Targeting the Myocardial Microenvironment: Novel Antiviral Strategies and Therapeutic Perspectives for Coxsackievirus B–Induced Myocarditis

**DOI:** 10.1161/JAHA.125.048926

**Published:** 2026-06-22

**Authors:** Yang Yue, Chengda Wei, Mengmeng Ding, Xiuwei Zhang, Xuemei Li, Lu Liu, Chengcheng Liu, Ce Wang

**Affiliations:** ^1^ Department of Orthodontics, School of Stomatology China Medical University Shenyang Liaoning China; ^2^ Department of Anesthesiology Shengjing Hospital of China Medical University Shenyang Liaoning China; ^3^ Department of Pathology The Fourth Affiliated Hospital of China Medical University Shenyang Liaoning China; ^4^ Department of Pediatrics Shengjing Hospital of China Medical University Shenyang Liaoning China; ^5^ Department of Preventive Dentistry, School and Hospital of Stomatology, Liaoning Provincial Key laboratory of Oral Diseases China Medical University Shenyang Liaoning China; ^6^ Department of Rehabilitation Shengjing Hospital of China Medical University Shenyang Liaoning China

**Keywords:** cardiomyocyte, Coxsackievirus B3, mechanism, microenvironment, myocarditis, Basic Science Research, Cell Therapy, Machine Learning, Translational Studies

## Abstract

Coxsackievirus B3 is a primary cause of viral myocarditis, with ≈20% of cases progressing to dilated cardiomyopathy. Despite its clinical significance, therapeutic strategies remain limited. The pathogenesis of coxsackievirus B3–induced myocarditis is complex, centered on the virus's ability to hijack the myocardial microenvironment, which is composed of various cell types and their interactions, influencing disease progression and outcomes. This review comprehensively summarizes the changes in fibroblasts, endothelial cells, mast cells, monocytes/macrophages, neutrophils, dendritic cells, natural killer cells, and lymphocytes within the myocardial microenvironment during coxsackievirus B3 infection, highlighting their critical roles in disease development. Additionally, it provides an overview of recent findings on potential therapeutic approaches targeting the myocardial microenvironment. Targeted regulation of cellular functions, key molecular pathway intervention, intelligent delivery system development, and noninvasive biomarker identification, combined with organoid and artificial intelligence technologies, pave the way for precision therapy and diagnosis centered on myocardial microenvironment remodeling.

**Nonstandard Abbreviations and Acronyms**
BMP7: bone morphogenetic protein 7
CAR: coxsackievirus and adenovirus receptor
CCL: chemokine (C‐C motif) ligand
CD: cluster of differentiation
CVB3: coxsackievirus B3
CXCL: chemokine (C‐X‐C motif) ligand
DC: dendritic cell
DCM: dilated cardiomyopathy
Drp1: dynamin‐related protein 1
EC: endothelial cell
IP‐10: interferon γ–induced protein 10
LC3: microtubule‐associated protein 1 light chain 3
MC: mast cell
MCP‐1: monocyte chemoattractant protein‐1
NF‐κB: nuclear factor‐κB
NK: natural killer
NLRP3: NOD‐like receptor family pyrin domain containing 3
SPP1: secreted phosphoprotein 1
TGF‐β: transforming growth factor‐β
Th: T helper
TLR: toll‐like receptor
TNF‐α: tumor necrosis factor‐α
Treg: regulatory T cell
VEGF: vascular endothelial growth factor
VMC: viral myocarditis
YAP: yes‐associated protein

Coxsackievirus B3 (CVB3) is the most important pathogen of viral myocarditis (VMC) worldwide. Its infection can trigger a progressive pathologic process, ranging from acute inflammation to chronic myocardial injury and even dilated cardiomyopathy (DCM).^1^ Although previous studies have mostly focused on the single dimension of direct viral damage to cardiomyocytes or host immune responses, and traditional therapeutic strategies mainly target viral replication or broad‐spectrum anti‐inflammatory interventions, their clinical efficacy is limited and lacks precise targeting. In recent years, the research paradigm has gradually shifted toward dissecting the dynamic interactions among multiple cell types in the cardiac microenvironment and their regulatory roles in disease progression. This perspective prospectively integrates host‐directed therapy and microenvironment reprogramming into the clinical translation pathway, which is significantly distinct from the fragmented analytical paradigm of existing literature.

In CVB3‐induced myocarditis, diverse cells within the myocardial microenvironment interact through complex signaling networks to collectively drive the pathologic process. As the primary target of the virus, cardiomyocyte injury directly initiates inflammatory responses and functional dysfunction. Cardiac fibroblasts not only maintain structural support and repair but have also been identified as an efficient “hidden reservoir” for CVB3 replication. The chemokine (C‐C motif) ligand (CCL) 20 secreted by cardiac fibroblasts recruits T helper (Th) 17 cell infiltration and exacerbates inflammation; meanwhile, the crosstalk between cardiac fibroblasts and mast cells leads to the release of CCL2/tumor necrosis factor (TNF)‐α, promoting the recruitment of monocytes/macrophages and fibrosis.^2^ Endothelial cells are affected early during viral invasion. Furthermore, recent single‐cell studies have revealed significantly enhanced vascular endothelial growth factor A (VEGFA)–mediated paracrine communication between endothelial cells (ECs) and Trem2^+^ (triggering receptor expressed on myeloid cells) reparative macrophages after infection, which regulates angiogenesis and tissue repair.^3^ For immune cells, natural killer (NK) cells guide macrophage polarization toward M1/M2 phenotypes by secreting interferon‐γ or interleukin‐4 in a sexually dimorphic manner, explaining the higher susceptibility in men^4^; B cells aggravate inflammation by inhibiting M2 polarization. In addition, dendritic cells, neutrophils, and other cell types are also involved in the biological processes of CVB3‐induced myocarditis. The above cellular network suggests that targeting specific cell–cell interaction nodes may represent a novel strategy for intervening in CVB3‐induced myocarditis.

Against the above research background, this review takes “the myocardial microenvironment as a core therapeutic target for CVB3 myocarditis” as the central theme. It systematically summarizes the composition and structural features of the myocardial microenvironment, as well as the regulatory mechanisms underlying its physiological homeostasis, and deeply analyzes the mechanisms linking microenvironment disorder to the progression of CVB3 myocarditis. Meanwhile, this review integrates novel antiviral strategies targeting the myocardial microenvironment in recent years, dissecting the mechanisms, advantages, and limitations of various strategies. Finally, it prospects future research directions and clinical translation prospects in this field, aiming to provide a comprehensive and systematic theoretical reference for subsequent basic research and clinical therapy targeting the myocardial microenvironment.

## THE ALTERATIONS AND ROLES OF THE MYOCARDIAL MICROENVIRONMENT IN CVB3 MYOCARDITIS

In CVB3‐induced myocarditis, dynamic communication among multiple cell types within the myocardial microenvironment profoundly influences disease progression. In the early stage of infection, cardiomyocytes, as the primary viral targets, are invaded by CVB3 and release chemokines, such as chemokine (C‐X‐C motif) ligand (CXCL) 10, to recruit natural killer cells, which subsequently suppress viral replication by secreting interferon‐γ, thereby exerting a critical antiviral role in the initial phase of infection. In addition, β‐arrestins expressed by cardiomyocytes can activate the cyclic GMP‐AMP synthase– stimulator of interferon genes pathway, promote interferon‐β production, and drive the expansion of immune cells in secondary lymphoid organs, thus amplifying local and systemic inflammatory responses.^5^ Endothelial cells activate the caspase activation and recruitment domain 8 inflammasome following CVB3 infection, triggering pyroptosis and amplifying local inflammation. This pathway also promotes viral transmission to adjacent cardiomyocytes, forming an “endothelium‐myocardium” cascade injury axis.^6^

As the disease progresses, interactions among immune cells dominate the balance between tissue injury and repair. Dendritic cells (DCs) in tolerant C57BL/6 mice secrete high levels of IP‐10 (interferon γ–induced protein 10) and regulated on activation, normal T‐cell expressed and secreted, drive the expansion of cluster of differentiation (CD) 4^−^/CD8^+^ cross‐presenting subsets, and promote Th1 polarization via toll‐like receptor (TLR) 3 signaling, thereby effectively controlling viral replication and alleviating myocardial injury. In contrast, DC function is dysregulated in TLR3‐deficient mice, shifting toward a Th17 response and exacerbating cardiac pathology.^7^ B cells aggravate inflammation by inhibiting M2 macrophage polarization: in B‐cell knockout models, myocardial pathologic scores are significantly reduced, and the M2 ratio is increased, whereas B‐cell adoptive transfer reverses this protective effect, indicating that the B‐cell–M2 axis is a key proinflammatory node.^8^ In addition, interleukin‐21 receptor signaling drives the production of anti‐adenine nucleotide translocase (ANT) autoantibodies by promoting T follicular helper (Tfh) cell activation and B‐cell proliferation, exacerbating immune‐mediated injury; blockade of this pathway markedly alleviates myocarditis.^1^

Fibroblasts are not merely structural support cells but also active immunomodulators. Single‐cell spatial omics has revealed that fibroblasts form dense ligand–receptor networks with macrophages and ECs in infected hearts, such as regulating neutrophil extracellular trap formation and apoptosis via CXCL1, collectively shaping the inflammatory microenvironment.^7^, ^9^ The crosstalk between macrophages and ECs is equally critical: single‐cell sequencing has revealed enhanced communication between Trem2^+^ reparative macrophages and ECs in infected hearts, with macrophage‐derived VEGFA promoting angiogenesis; macrophage depletion or VEGF receptor inhibition results in impaired vascular repair and deteriorated cardiac function.^3^ Stabilin‐1, expressed on the surface of macrophages, mediates their adhesion to fibronectin and migration to injured regions, forming a protective barrier; its deficiency leads to excessive CD3^+^ T‐cell infiltration, reduced macrophage recruitment, and a significantly increased mortality rate.^10^

**Figure 1:**
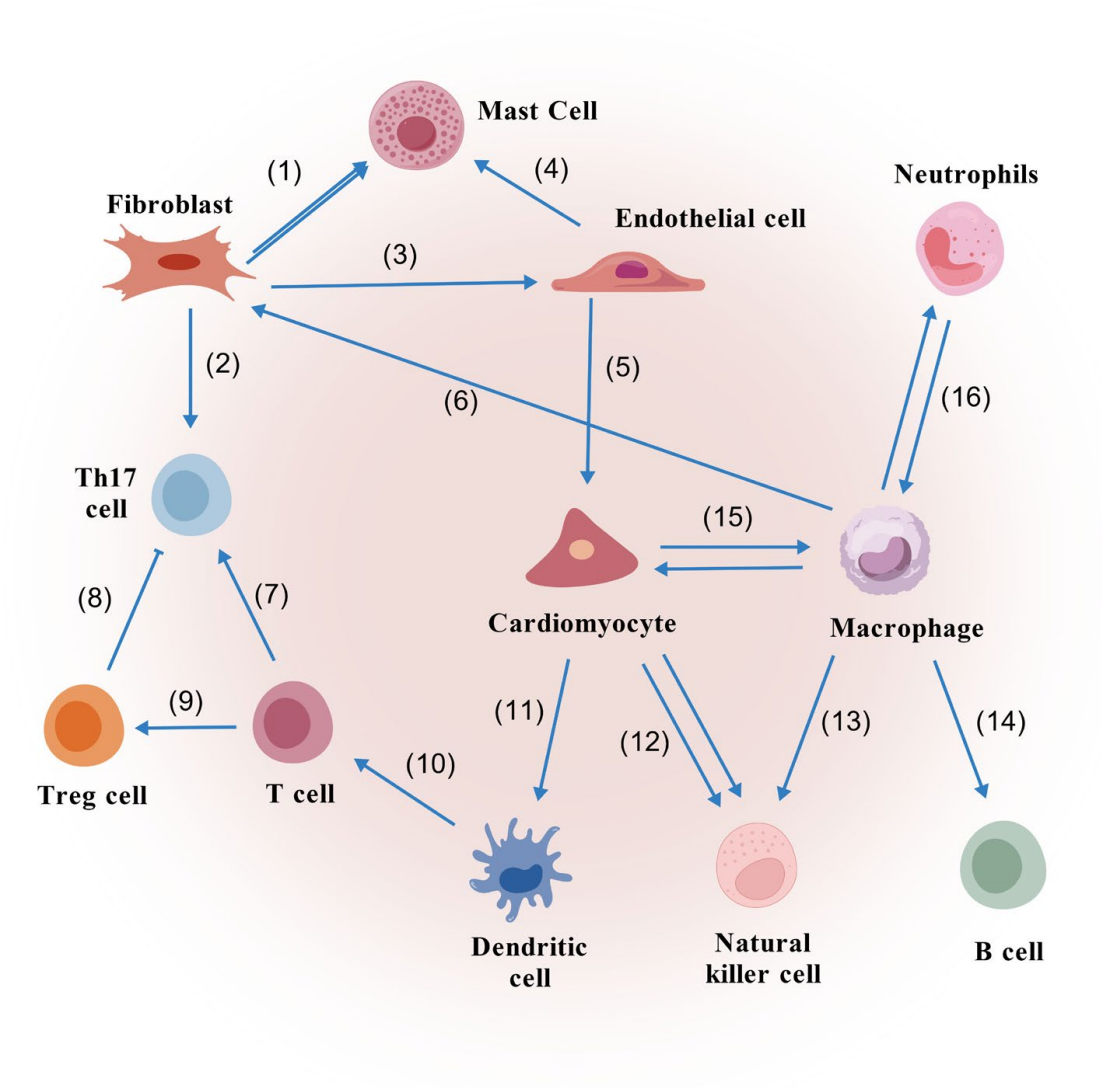
Intercellular communication and interaction mechanisms in the myocardial microenvironment. (1) Cardiac fibroblasts and mast cells interact to promote the release of inflammatory factors and chemokines. (2) Cardiac fibroblasts secrete chemokines to recruit Th17 cells. (3) Trem2^+^ reparative macrophages secrete paracrine factors to act on endothelial cells, which provide a reparative microenvironment for them. (4) NK cells secrete cytokines to regulate the polarization direction of macrophages. (5) B cells inhibit M2 polarization of macrophages through cell–cell contact or cytokine secretion. (6) Cardiomyocytes secrete chemokines to recruit NK cells, and NK cells kill infected cardiomyocytes. (7) Cardiomyocytes release damage‐associated molecules to activate macrophages. (8) Macrophages secrete factors to activate cardiac fibroblasts, which secrete chemokines to recruit monocytes. (9) Endothelial cells undergo pyroptosis to release viruses and inflammatory factors that spread to cardiomyocytes. (10) Dendritic cells present antigens to activate T cells and regulate the differentiation direction of T‐cell subsets. (11) Neutrophils secrete cytokines to recruit macrophages, which promote the activation of neutrophils. (12) Cardiac fibroblasts, endothelial cells, and macrophages form a ligand‐receptor network to synergistically regulate the inflammatory microenvironment. (13) Cardiomyocytes release antigens for uptake by dendritic cells, which activate and initiate adaptive immunity. (14) Treg cells secrete anti‐inflammatory factors to inhibit Th17 cell activation, and CVB3 infection disrupts this balance. (15) Endothelial cells undergo endothelial‐mesenchymal transition into myofibroblast‐like cells, which synergize with cardiac fibroblasts to promote fibrosis. (16) Mast cells degranulate to release activating factors that activate endothelial cells. NK indicates natural killer; Th17, T helper 17 cell; and Treg, regulatory T cell.

These mechanisms provide multidimensional targets for clinical intervention: targeting CXCL10 or enhancing NK cell function can strengthen early antiviral defense^11^; regulating DC–T‐cell polarization (eg, TLR3 agonists) or blocking the interleukin‐21 receptor/Tfh/B‐cell axis can suppress autoimmune injury^1^, ^12^; promoting M2 polarization or inhibiting B‐cell activity is expected to alleviate inflammation^8^; and intervening in endothelial‐to‐mesenchymal transition (eg, BMP7 [bone morphogenetic protein 7]) or enhancing macrophage–endothelial reparative crosstalk (eg, stabilin‐1 mimetics) may improve tissue remodeling.^3^, ^13^ Figure 1 illustrates the interactive network of various cells in the myocardial microenvironment.

It should be emphasized that the above findings and mechanisms are predominantly derived from preclinical studies, and further clinical validation and translation are required before their application in patient treatment. In recent years, explorations using human viral myocarditis samples have gradually revealed the clinical relevance of intercellular communication within the myocardial microenvironment during disease progression. Spatial transcriptomics, single‐cell sequencing, and immunohistochemical analyses based on patient endomyocardial biopsies, peripheral blood, or autopsy specimens have provided critical clues.

For example, in endomyocardial biopsy samples from patients with immune checkpoint inhibitor–associated myocarditis, single‐cell RNA sequencing has revealed a marked expansion of CXCL9^+^ fibroblasts and atypical chemokine receptor 1^+^ ECs, which shape the local inflammatory niche by secreting proinflammatory chemokines and drive CD8^+^ exhausted T‐cell infiltration.^1^ In addition, studies on human viral myocarditis autopsy tissues have shown upregulated expression of stabilin‐1 on the surface of CD68^+^ macrophages, and its deficiency is significantly associated with excessive CD3^+^ T‐cell infiltration and poor prognosis.^14^ During the acute phase of viral myocarditis, a strong ligand–receptor interaction exists between the secreted phosphoprotein 1 (SPP1)^+^ (osteopontin‐positive) macrophage subset and cardiac fibroblasts, and activation of this pathway has also been observed in human samples, suggesting that targeting SPP1 or its downstream signaling may represent a novel strategy to alleviate myocardial inflammation. Meanwhile, functional studies based on human peripheral blood and myocardial tissues have demonstrated that the proportion of interleukin‐10–secreting regulatory B cells dynamically changes in viral myocarditis, and prostaglandin E2 can promote their expansion, thereby inhibiting Th17 differentiation and attenuating myocardial injury.^15^

Collectively, the myocardial microenvironment, as a highly dynamic and multicellular signaling hub, plays a central role in the initiation and progression of viral myocarditis. However, the vast majority of current mechanistic insights remain at the stage of animal experiments. There is an urgent need to conduct more clinical studies using human samples to validate the applicability, temporal specificity, and interindividual heterogeneity of the above pathways in real‐world patients.

## CARDIOMYOCYTES

CVB3 infection of cardiomyocytes is the core pathogenic event in viral myocarditis. The virus enters cells by binding capsid proteins to cardiomyocyte surface receptors: coxsackievirus and adenovirus receptor (CAR) and decay‐accelerating factor. CAR not only mediates viral internalization but also activates downstream cascades, like p38 mitogen‐activated protein kinase, potentially triggering inflammation even without intact virions.^16^ A complete kinetic model of CVB3 infection has been established; on entry, CVB3 releases its positive‐sense RNA genome, which undergoes cap‐independent translation via an internal ribosome entry site. Meanwhile, the viral 2A protease cleaves host eukaryotic translation initiation factor 4G, suppressing cap‐dependent host translation to redirect resources for viral replication, causing metabolic dysregulation and stress.^17^ Double‐stranded RNA produced by viral replication can be recognized by retinoic acid–inducible gene I and TLR3, inducing the production of interferons and proinflammatory cytokines.^17^

With infection progression, sustained viral replication and immune activation promote nucleotide‐binding oligomerization domain–like receptor family pyrin domain containing 3 (NLRP3) inflammasome assembly. Caspase‐1–mediated gasdermin D cleavage triggers pyroptosis. Additionally, viral 2B protein disrupts calcium homeostasis, inducing mitochondrial Ca^2+^ overload and activating the Bax/Bcl‐2 apoptotic pathway, amplifying myocardial injury.^18^

Pyroptosis and apoptosis do not occur independently. Factors, such as interleukin‐1β, released during pyroptosis not only exacerbate local inflammation but may also promote mitochondrial stress and Bax activation in adjacent cells through paracrine mechanisms, thereby synergistically inducing apoptosis. Conversely, changes in mitochondrial membrane permeability and cytochrome *c* release during apoptosis may also activate the NLRP3 inflammasome, forming a positive feedback loop.

**Figure 2:**
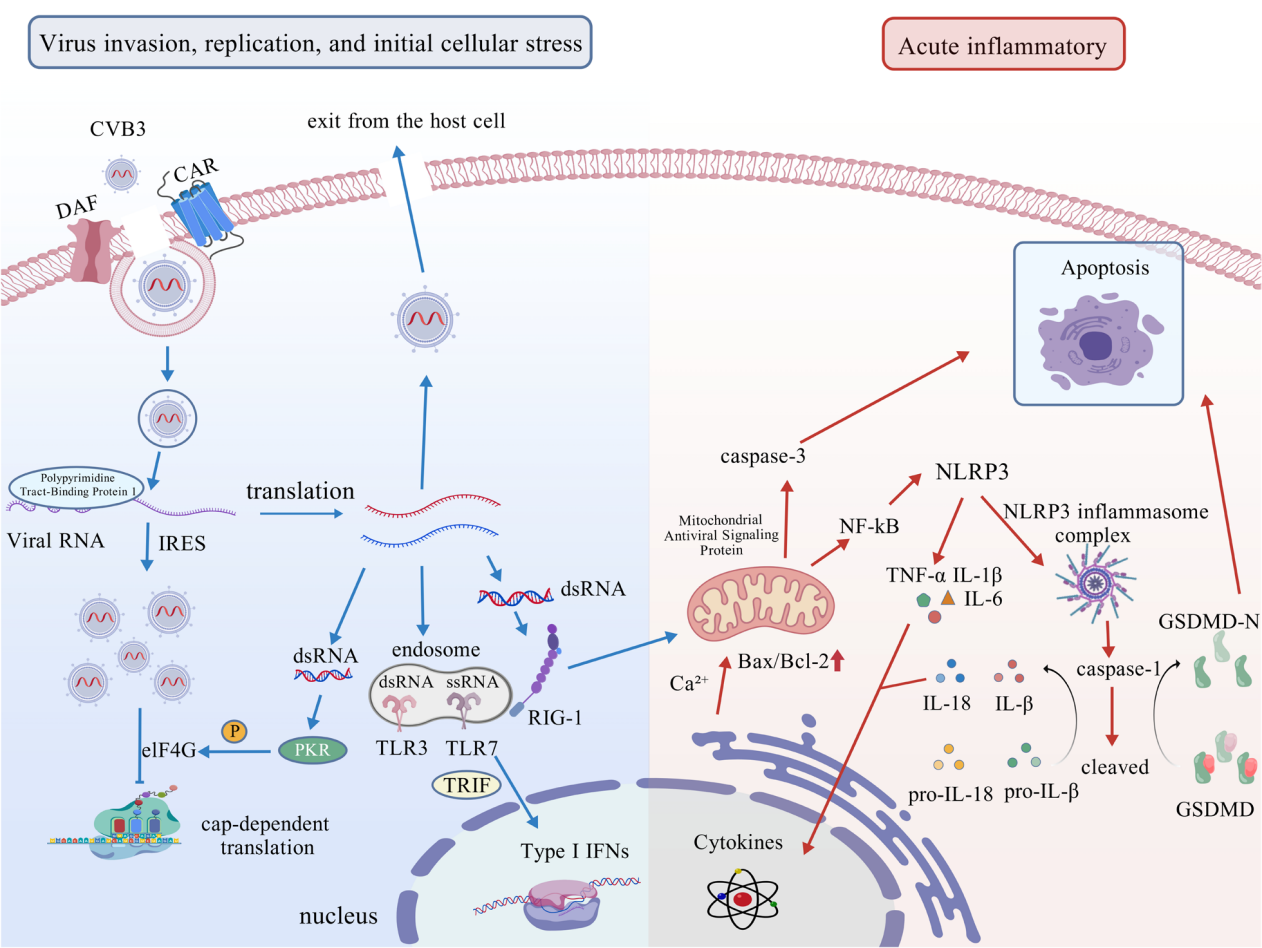
Key molecular mechanisms and pathologic progression of myocarditis induced by coxsackievirus B3. CVB3 invades host cells via DAF and CAR receptors, translates viral proteins through IRES elements, and inhibits host translation; viral dsRNA activates RIG‐I, TLR3/7, and other receptors as well as the NF‐κB pathway, inducing the expression of type I interferons and cytokines. Sustained NF‐κB activation promotes the release of proinflammatory cytokines, and NLRP3 inflammasome activates caspase‐1 to trigger pyroptosis and produce mature cytokines; Bax/Bcl‐2 imbalance initiates the mitochondrial apoptotic pathway, activating caspase‐3 to mediate cardiomyocyte apoptosis. CAR indicates coxsackievirus and adenovirus receptor; CVB3, coxsackievirus B3; DAF, decay‐accelerating factor; dsRNA, double‐stranded RNA; eIF4G, eukaryotic translation initiation factor 4G; GSDMD, gasdermin D; IRES, internal ribosome entry site; NF‐κB, nuclear factor‐κB; NLRP3, NOD‐like receptor family pyrin domain containing 3; RIG‐I, retinoic acid–inducible gene I; TLR, toll‐like receptor; and TNF‐α, tumor necrosis factor‐α.

Histologically, widespread necrosis, interstitial edema, and dense mononuclear/lymphocytic infiltration are observed; functionally, left ventricular ejection fraction decreases and diastolic function impairs. Serum creatine kinase‐MB and cardiac troponin I (cTnI) remain elevated, correlating positively with interleukin‐1β, TNF‐α, and interleukin‐6 concentrations. Figure 2 illustrates CVB3‐induced biological mechanisms in cardiomyocytes.

CVB3 infection triggers enhanced glycolysis in cardiomyocytes, and the intermediates generated by activated aerobic glycolysis support viral replication. Meanwhile, the virus impairs mitochondrial function via Drp1 (dynamin‐related protein 1)–dependent excessive mitochondrial fission, accompanied by decreased activities of succinate dehydrogenase and cytochrome *c* oxidase, reduced mitochondrial membrane potential, and ultimately diminished ATP synthesis.^19^ Additionally, CVB3 infection dysregulates the creatine kinase system, with decreased mitochondrial creatine kinase and cytoplasmic creatine kinase activities that correlate positively with viral load and levels of inflammatory cytokines; the results were validated in myocardial biopsy samples from mice and patients with chronic inflammatory heart disease.^20^

All the above mechanisms have been verified in animal experiments. Human myocardial biopsy data also corroborate the elevated expression of nucleotide‐binding oligomerization domain 2 mRNA in CVB3‐positive patients, which promotes interleukin‐1β release and cardiomyocyte pyroptosis by activating the NLRP3 inflammasome, consistent with animal models. Although large‐scale single‐cell RNA‐sequencing data are lacking, biopsies have confirmed key molecular events, including upregulation of Bax and activation of caspase‐1 in human myocarditis lesions.^21^, ^22^

These mechanisms provide multiple targets for related therapies. Targeting the CAR receptor inhibits viral entry, and combined use with siRNA synergistically reduces viral titers^23^; blockade of the nucleotide‐binding oligomerization domain 2–NLRP3–caspase‐1–gasdermin D axis alleviates myocardial inflammation^21^; the Drp1 inhibitor Mdivi‐1 and astragaloside IV regulate mitochondrial dynamics and inhibit apoptosis, respectively.^19^ Furthermore, targeting the viral hijacking of glycolysis can restore myocardial energy homeostasis.

## FIBROBLASTS

In addition to cardiomyocytes, cardiac fibroblasts have been identified as another important site for CVB3 replication. In a mice model, during the early stages of CVB3 infection, the viral replication rate in fibroblasts is higher than in cardiomyocytes.^24^

Autophagy, a highly conserved catabolic process, can influence the prognosis of VMC by regulating viral replication and release, and modulating immune responses and cell apoptosis. Studies have shown that CVB3 infection induces significant autophagic activity in cardiac fibroblasts.^25^ Similar to CVB3‐infected fibroblasts, transfection of cardiac fibroblasts with plasmid MKS1 (pMKS1) containing the full‐length CVB3 cDNA results in punctate LC3 (microtubule‐associated protein 1 light chain 3) fluorescence, indicating that autophagosome formation is a consequence of CVB3 replication.^25^ Furthermore, reducing autophagy in CVB3‐infected fibroblasts from mice ventricles using the autophagy inhibitors 3 methyladenine (3‐MA) or autophagy‐related gene (Atg) 7‐siRNA leads to a decrease in proinflammatory cytokines interleukin‐6 and TNF‐α, which helps protect the heart.^25^

Single‐cell RNA sequencing shows that in CVB3‐induced murine myocarditis models, cardiac fibroblasts have heterogeneous expression patterns related to inflammation and immune regulation.^26^

Fibroblasts overproduce and release extracellular matrix components, including transforming growth factor‐β (TGF‐β), and are involved in various pathologic states, particularly fibrosis and tissue remodeling. The results from mice studies indicate that CVB3 infection activates signaling pathways in cardiac fibroblasts, such as the TGF‐β1/Smad2/3 pathway, promoting the synthesis of collagen types I and III and exacerbating cardiac fibrosis.^27^

Notably, mechanical stress can exert a synergistic effect with viral infection, further exacerbating fibroblast activation and fibrosis progression. In the context of CVB3 infection, the viral‐induced inflammatory response can inherently activate fibroblasts; when superimposed with abnormal cardiac mechanical loading (such as hypertension or ventricular dilation), mechanical stress significantly accelerates fibrosis progression through a positive feedback loop between metabolic reprogramming and inflammatory factor release. This “virus‐mechanics” dual stimulation model may explain why some patients with VMC are more prone to developing DCM. Experiments have demonstrated that cyclic stretch can significantly enhance the stimulatory effect of TGF‐β1 on collagen synthesis in fibroblasts, whereas inhibition of mechanosensitive pathways (such as rho‐associated coiled‐coil containing protein kinase or YAP [yes‐associated protein]) can partially reverse this effect.^28^

**Figure 3:**
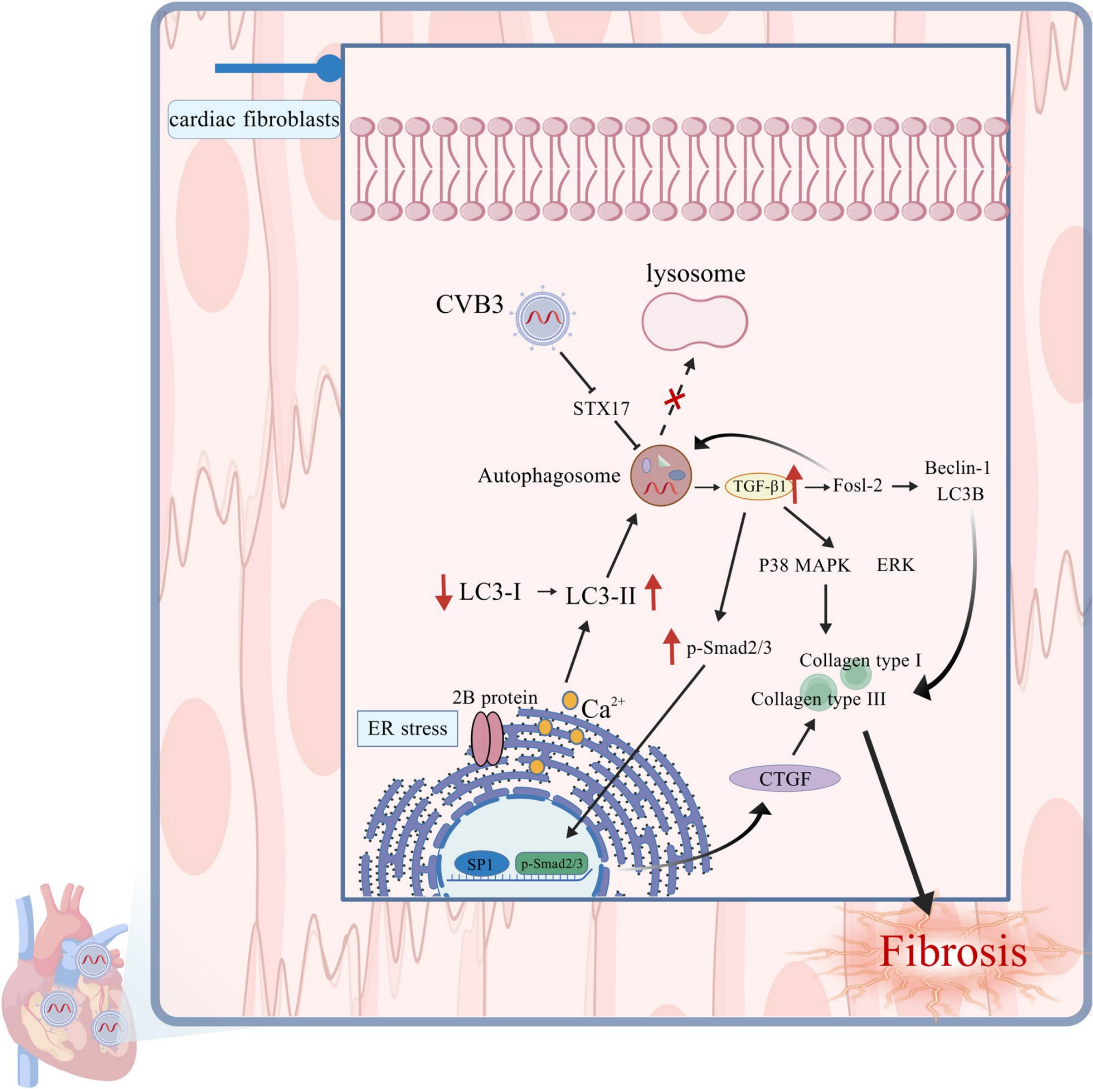
Molecular mechanisms of CVB3 infection in cardiac fibroblasts. CVB3 enters cardiac fibroblasts and its 2B protein induces ER stress via Ca^2+^ dysregulation, promoting LC3‐I conversion to LC3‐II and autophagosome formation. Reduced STX17 expression blocks autophagosome‐lysosome fusion, leading to autophagic flux impairment. TGF‐β1 activates downstream signaling pathways, including p38 MAPK, ERK, and Smad2/3, whereas Fosl‐2 upregulates beclin‐1 and LC3B to enhance autophagy. These events synergistically drive the synthesis of collagen type I/III and CTGF expression, ultimately contributing to cardiac fibrosis. CTGF indicates connective tissue growth factor; CVB3, coxsackievirus B3; ER, endoplasmic reticulum; ERK, extracellular signal‐regulated kinase; LC3, microtubule‐associated protein 1 light chain 3; MAPK, mitogen‐activated protein kinase; STX17, syntaxin 17; and TGF‐β, transforming growth factor‐β.

Current strategies to block fibrosis focus on inhibiting profibrotic factors. TGF‐β is a primary driver of fibrosis, but its inhibition is associated with adverse effects because of its pleiotropic actions. Based on these studies, precise targeting of fibroblast‐derived substances is critical. Figure 3 illustrates additional details about the molecular mechanisms underlying CVB3 infection of fibroblasts.

## ENDOTHELIAL CELLS

ECs serve as a barrier against circulating inflammatory cells. Under homeostatic conditions, inflammation is tightly controlled by the endothelial barrier; however, proinflammatory conditions can lead to EC inflammation and allow immune cell infiltration.

Single‐cell sequencing analysis in mice has revealed that ECs are a major component of the infiltrate in hearts infected with CVB3. In the infected state, ECs exhibit upregulated expression of specific transcription factors, such as activating transcription factor 4, interferon regulatory factor 1, and signal transducer and activator of transcription 6. Conversely, survival and angiogenesis‐related factors, like Myc and nuclear factor erythroid 2–related factor 2, are downregulated.^29^

The coreceptor CAR on ECs is crucial for maintaining vascular permeability and tight junction stability. Park and colleagues developed an endothelium‐specific CAR knockout mice model and observed that the lack of CAR led to impaired endothelial barrier function, characterized by increased transmembrane resistance and enhanced CVB3 permeability.^30^ Restoring CAR receptor function or stabilizing tight junctions may help rebuild the vascular barrier, thereby limiting inflammatory cell infiltration and viral spread. Another study found that CVB3 infection of myocardial microvascular ECs activates fractalkine via the extracellular signal‐regulated kinase 1/2 signaling pathway, promoting viral infection.^31^ When fractalkine expression was inhibited using RNA interference or extracellular signal‐regulated kinase 1/2 signaling was blocked, the replication efficiency of CVB3 was reduced.

ECs can undergo endothelial‐to‐mesenchymal transition, differentiating into myofibroblast‐like cells, which promotes cardiac fibrosis. Studies at the cellular and animal levels have confirmed that concurrent evidence indicates that after CVB3 infection, there is colocalization of endothelial markers (CD31 and vascular endothelial–cadherin) with mesenchymal markers (FSP1 [Fibroblast‐specific protein 1] and α‐smooth muscle actin), indicating a transition from ECs to mesenchymal cells.^32^, ^33^ DCM is a poor outcome of CVB3‐induced myocarditis, suggesting that endothelial‐to‐mesenchymal transition may play a role in the progression from VMC to DCM. In summary, ECs play a critical role in CVB3‐induced myocarditis, both in direct involvement in viral invasion and proliferation and in the dysregulation of local immune responses and maintenance of vascular integrity.

## CARDIAC MAST CELLS

Mast cell (MC) innate immune cells are widely distributed in cardiovascular tissues, such as the adventitia and interstitial myocardium. They participate in the development and progression of cardiovascular diseases by releasing prestored or newly synthesized mediators, including histamine, tryptase, and cytokines. Researchers using 2 MC‐deficient mice models found that these mice had significantly higher survival rates and less myocardial necrosis and cellular infiltration when infected with encephalomyocarditis virus compared with control groups.^34^ Another study showed that in a viral‐induced heart failure mice model, the gene expression levels of MC chymase and tryptase began to increase from day 5 after infection and continued until day 14, coinciding with increased inflammation, extensive myocardial necrosis, and fibrosis.^35^

Another characteristic of MCs is their strategic location around blood vessels, allowing them to regulate EC function. MCs synthesize various EC activators, including platelet‐activating factor, interleukin‐1β, interleukin‐4, and TNF‐α. Multiple studies have shown that MC degranulation is a major source of TNF‐α. Recent research by Luo et al^2^ revealed the role of the stem cell factor/MC/CCL2/monocyte/macrophage axis in promoting CVB3‐induced myocarditis and cardiac fibrosis. Specifically, the interaction between MCs and fibroblasts promoted the production of chemokines, like CCL2, and proinflammatory mediators, like TNF‐α, exacerbating inflammation and fibrosis in the heart.

Efforts to mitigate CVB3‐induced inflammation and ventricular remodeling by inhibiting MC activation are promising. For example, the histamine H1 receptor antagonist bepotastine improves cardiac function in CVB3‐infected VMC mice.^36^ Although MC inhibition shows potential for VMC, its role in the chronic phase of DCM remains unclear. The specific functions and interaction networks of MCs across viral infection stages, plus individual variations in MC reactivity, may affect disease progression. In a CVB3‐induced myocarditis mice model, females displayed higher MC activation and fibrosis, whereas males showed greater complement activation and increased macrophages/neutrophils.^37^ Thus, although CVB3 infection generally enhances cardiac MC activity, the underlying immune mechanisms may differ by gender.

## MONOCYTES AND MACROPHAGES

**Figure 4:**
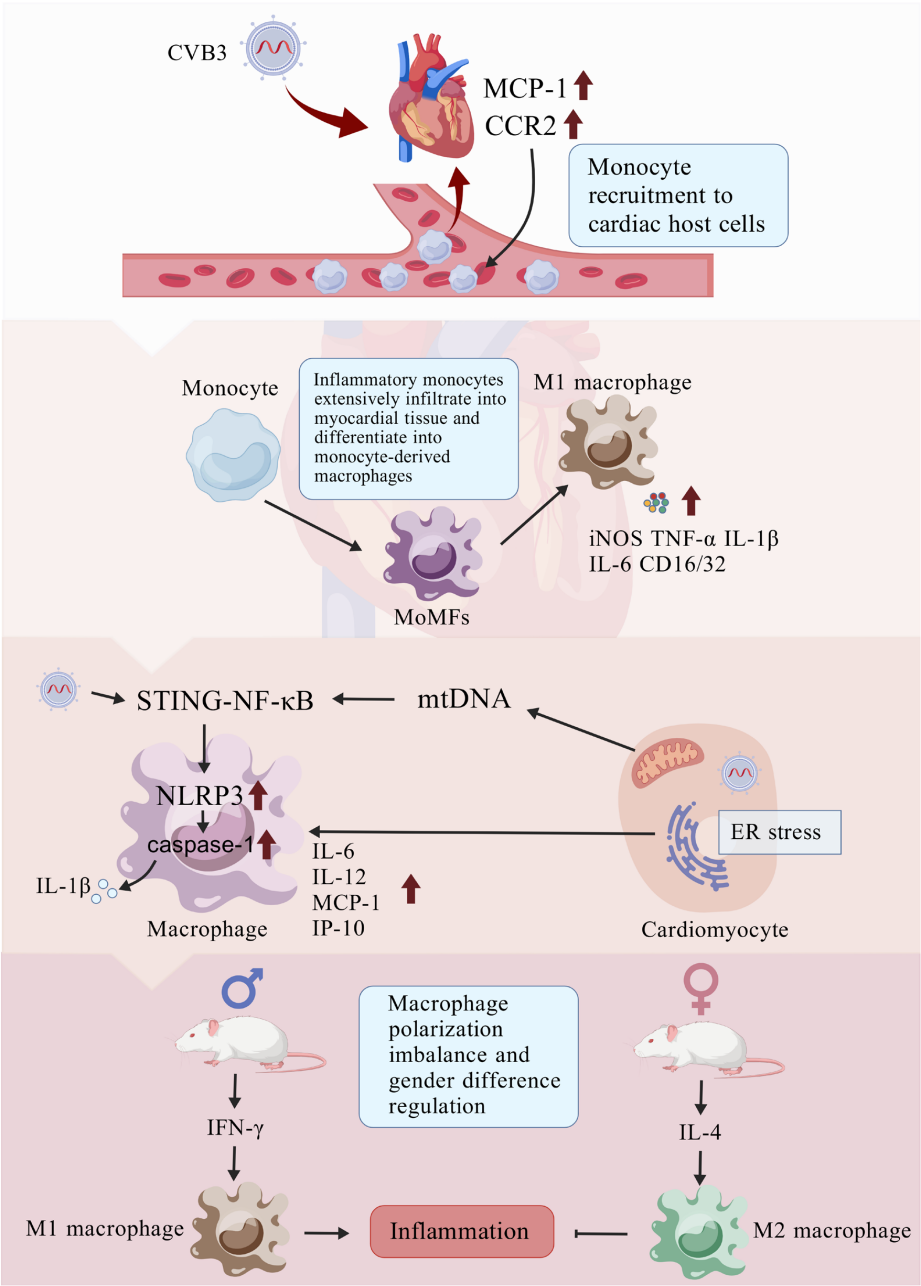
Macrophage molecular mechanisms and dynamic changes during CVB3‐induced cardiac infection. CVB3 infects cardiomyocytes and induces MCP‐1 release, which recruits CCR2^+^ monocytes to cardiac tissue via the MCP‐1/CCR2 axis. Recruited monocytes differentiate into MoMFs that polarize to proinflammatory M1 phenotype, accompanied by reduced resident macrophages. CVB3 capsid proteins (VP1/VP2) and myocardial mtDNA activate NLRP3 inflammasome and STING–NF‐κB pathway, respectively, whereas transmissible ER stress enhances cytokine secretion. Macrophage M1/M2 polarization imbalance persists with gender differences: IFN‐γ drives M1 in males, and IL‐4 promotes M2 in females. CCR indicates C‐C chemokine receptor; CVB3, coxsackievirus B3; ER, endoplasmic reticulum; iNOS, inducible nitric oxide synthase; IFN‐γ, interferon‐γ; IL, interleukin; MCP‐1, monocyte chemoattractant protein‐1; MoMF, monocyte‐derived macrophage; mtDNA, mitochondrial DNA; NF‐κB, nuclear factor‐κB; NLRP3, NOD‐like receptor family pyrin domain containing 3; STING, stimulator of interferon genes; and TNF‐α, tumor necrosis factor‐α.

Cardiac macrophages can be categorized into 2 main types: embryonically derived resident macrophages and monocyte‐derived macrophages. In a healthy state, these 2 populations serve distinct physiological functions. Following viral infection, there is a significant infiltration of monocytes into the heart. On entering the damaged cardiac tissue, monocytes undergo phenotypic and functional changes, participating in both inflammatory injury and tissue repair processes. A study has indicated that by inhibiting C‐C chemokine receptor 2 in mice, the number of monocyte‐derived macrophages can be reduced, thereby alleviating the symptoms of acute myocarditis and promoting the recovery of resident macrophage numbers.^38^ This suggests that modulating the balance between different macrophage types may offer new therapeutic strategies. Figure 4 illustrates the activities and mechanisms of macrophages during CVB3 infection.

Single‐cell sequencing results from mice revealed that macrophages are not only the first inflammatory cells recruited to the heart but also constitute the major infiltrating cell subpopulation in the early stages of VMC.^39^ In addition to clearing and presenting damaged cardiomyocytes to initiate adaptive immune responses, macrophages regulate the cardiac immune microenvironment through the production of cytokines, chemokines, and growth factors, playing a crucial role in the initiation, amplification, and maintenance of inflammation.

The mechanisms by which CVB3 affects macrophage activation are complex and not fully understood. The results from the mice study indicate that NLRP3 inflammasome is upregulated not only in cardiomyocytes but also in infiltrating macrophages during CVB3 infection. The CVB3 capsid proteins VP1 and VP2 can effectively activate the NLRP3 inflammasome in macrophages, contributing to the development of VMC.^40^ Furthermore, another study using a mice model demonstrated that CVB3 infection leads to the release of mitochondrial DNA from cardiomyocytes, which activates the stimulator of interferon genes signaling pathway in macrophages, triggering an inflammatory response.^41^ Interestingly, this crosstalk is also evident in endoplasmic reticulum stress. In mice after CVB3 infection, endoplasmic reticulum stress in infiltrating macrophages is significantly enhanced, leading to the production of proinflammatory cytokines, such as interleukin‐6, interleukin‐12, MCP‐1 (monocyte chemoattractant protein‐1), and IP‐10.^42^ This suggests that blocking endoplasmic reticulum stress transmission could be a new therapeutic strategy for VMC.

Macrophages can be polarized into classically activated M1 or alternatively activated M2 phenotypes based on the cardiac environment. M1 macrophages secrete proinflammatory factors, such as interleukin‐1β and TNF‐α, whereas M2 macrophages produce anti‐inflammatory factors, like arginase‐I and interleukin‐10, promoting tissue repair and wound healing.

Single‐cell sequencing has revealed a multidirectional crosstalk network among macrophages, fibroblasts, and ECs. Recent studies indicate that during the acute phase of CVB3 infection, a subset of macrophages highly expressing Spp1 (osteopontin) exists in the heart. These macrophages activate fibroblasts by secreting SPP1, prompting them to highly express CCL2/CCL7, which, in turn, recruits more monocytes into the heart, forming a positive feedback loop for inflammatory amplification. Inhibition of SPP1 significantly reduces macrophage infiltration, alleviates inflammation and myocardial injury, and improves cardiac function.^43^ Concurrently, another published single‐cell RNA‐sequencing study further delineated the dynamic crosstalk between macrophages and ECs. The study found that Trem2^+^ reparative macrophages are the major infiltrating population during the peak of infection (day 7), and the VEGFA they secrete promotes capillary endothelial proliferation and angiogenesis. Either macrophage depletion or VEGF receptor inhibition leads to decreased VEGFA levels, suppressed endothelial proliferation, and exacerbated cardiac dysfunction.^3^

Additionally, metabolic reprogramming regulates macrophage polarization and migration. In the CVB3‐infected mice model, a study first revealed that methylenetetrahydrofolate dehydrogenase 2 (a key enzyme in 1‐carbon metabolism) is significantly upregulated in macrophages following CVB3 infection and that it inhibits macrophage polarization toward the proinflammatory M1 phenotype. Either supplementation of methylenetetrahydrofolate dehydrogenase 2 or a high‐folate diet attenuates myocarditis, indicating metabolic intervention as a novel therapeutic approach.^44^ Furthermore, CVB3 inhibits the autophagic degradation of argininosuccinate synthetase 1 through its capsid protein VP2, upregulates argininosuccinate synthetase 1, activates urea cycle metabolic reprogramming, and drives macrophage conversion toward the proinflammatory M1 type.^45^ Supplementation of citrulline reverses argininosuccinate synthetase 1 upregulation, restores the anti‐inflammatory phenotype of macrophages, and alleviates myocardial injury.^45^

## NEUTROPHILS

Neutrophils, as part of the innate immune system, rapidly accumulate in cardiac tissue during the early stages of CVB3 infection. The results of gene expression analysis and single‐cell sequencing in mice show that after CVB3 infects cardiomyocytes, it activates the NF‐κB and interferon regulatory factor signaling pathways through pattern recognition receptors, inducing the secretion of chemokines, such as CCL2, CXCL1, and CXCL2/3. Among these, the CXCL2/CXCL3–chemokine (C‐X‐C motif) receptor 2 axis drives the recruitment of neutrophils from the peripheral blood to the heart. Importantly, neutrophils themselves can secrete CXCL2/3, forming an “autocrine recruitment loop” that leads to a dramatic accumulation of neutrophils in the myocardium around 4 days after infection.^46^, ^47^

At 4 to 7 days after viral infection in mice, neutrophils infiltrating the heart recognize CVB3 RNA via endosomal TLR8, which activates inflammatory pathways. This results in the upregulation of the surface adhesion molecule CD11b, enhanced adhesive capacity, and the secretion of proinflammatory cytokines to recruit macrophages and amplify the inflammatory response.^48^ Meanwhile, activation of the interleukin‐12/signal transducer and activator of transcription 4/interferon‐γ axis enhances neutrophil function to restrict viral replication.^49^

From 7 to 14 days after infection, neutrophils release neutrophil extracellular traps. Although neutrophil extracellular traps can physically trap viruses, they exacerbate tissue damage by activating TLR9 and the complement system, a process promoted by TNF‐α.

When neutrophils are depleted using specific antibodies, such as 1A‐8 (anti‐Ly6G), there is no significant impact in mice on viral infection, disease susceptibility, or histopathologic changes in the heart and pancreas.^50^

**Figure 5:**
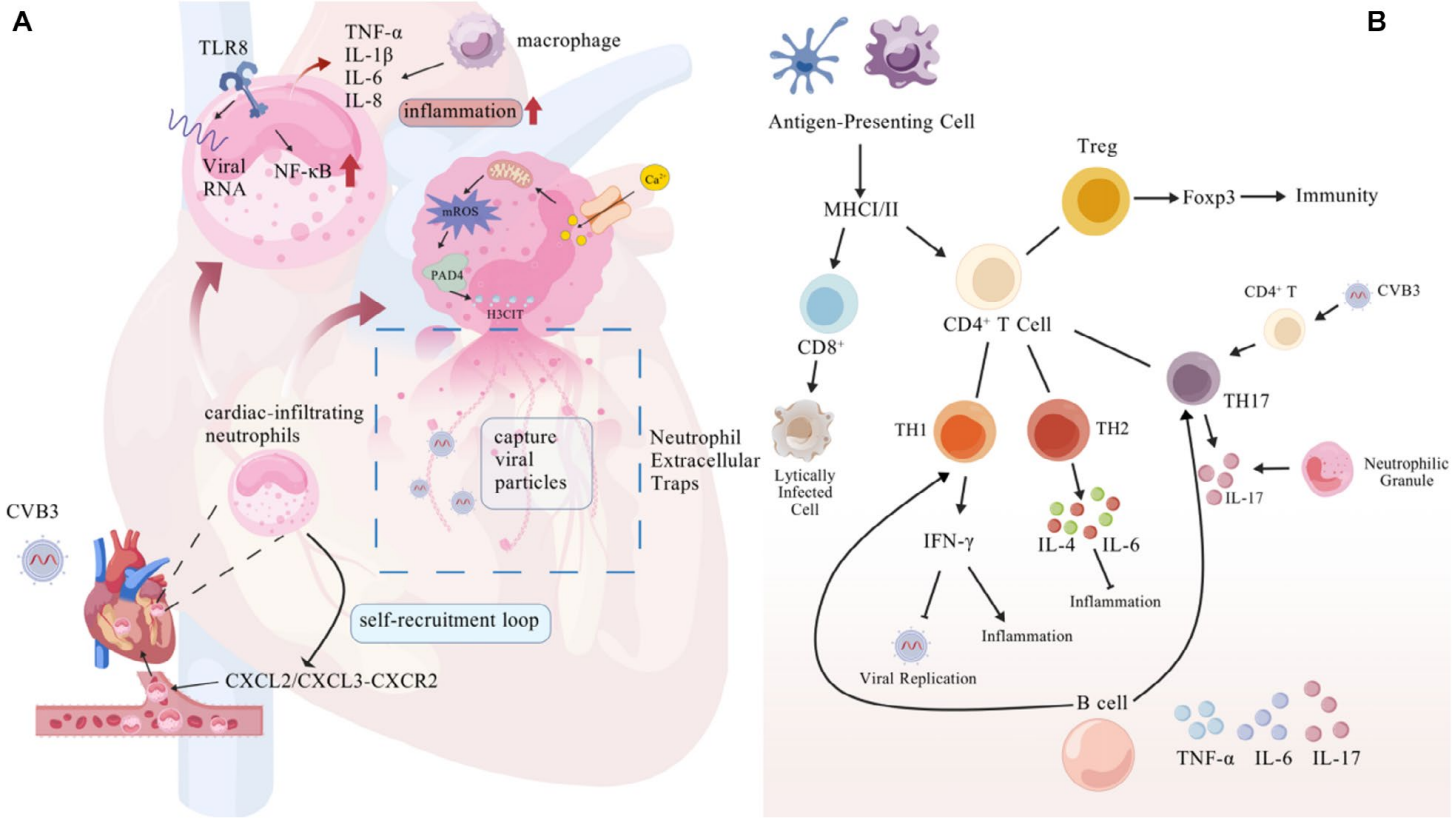
Neutrophil‐ and lymphocyte‐mediated immune mechanisms in CVB3‐induced myocarditis. This schematic integrates innate and adaptive immune responses in CVB3‐induced myocarditis, showing neutrophil‐driven inflammation (**A**) and lymphocyte‐mediated immune regulation (**B**). **A**, CVB3 activates cardiac neutrophils via TLR8/NF‐κB signaling to produce proinflammatory cytokines, with self‐recruitment via CXCL2/CXCL3‐CXCR2. Viral RNA induces mROS‐PAD4–dependent NET release to capture viruses and amplify inflammation. **B**, Antigen‐presenting cells activate CD4^+^/CD8^+^ T cells and B cells. CD4^+^ T cells differentiate into Th1/Th2/Th17/Treg subsets, secreting cytokines to regulate viral replication, inflammation, and immune balance. CVB3 indicates coxsackievirus B3; CXCL, chemokine (C‐X‐C motif) ligand; CXCR, chemokine (C‐X‐C motif) receptor; IFN‐γ, interferon‐γ; IL, interleukin; MHC, major histocompatibility complex; mROS‐PAD, mitochondrial reactive oxygen species‐peptidylarginine deiminase; NET, neutrophil extracellular trap; NF‐kB, nuclear factor‐kB; Th, T helper cell; TLR, toll‐like receptor; TNF‐α, tumor necrosis factor‐α; and Treg, regulatory T cell.

Interleukin‐37 has been shown to mitigate CVB3‐induced myocardial injury in mice by inhibiting the formation of neutrophil extracellular traps, thereby reducing inflammation and improving cardiac function.^51^ In summary, although neutrophils themselves do not directly control viral replication, they exhibit a “double‐edged sword” role in CVB3‐induced myocarditis: they limit viral spread in the early phase, but excessive activation and neutrophil extracellular trap release lead to myocardial injury. Specific neutrophil subsets participate in tissue repair in the late phase, and they may interact with other immune cells, such as monocytes/macrophages, to modulate the intensity of the inflammatory response, promote tissue repair, and influence fibrosis,^50^, ^51^ providing an important basis for stage‐specific targeted interventions. Figure 5A illustrates the mechanism of neutrophils following CVB3 infection.

## DENDRITIC CELLS

DCs are potent antigen‐presenting cells that activate both innate and adaptive immune responses following viral infections. In CVB3‐infected mice models, the number of bone marrow–derived myeloid DCs increases, and these cells exhibit a more mature phenotype.^52^ Most studies on cardiac DCs have focused on their roles in transplant tolerance and autoimmune myocarditis, where DC activation is a clear pathogenic factor. In the context of VMC, impaired DC function or maturation can lead to adverse outcomes. For example, DCs from C57BL/6 mice, which are relatively resistant to CVB3, secrete higher levels of proinflammatory cytokines and chemokines compared with those from the susceptible A.BY/SnJ strain.^53^ This indicates that specific types of DCs may protect against CVB3 infection by regulating the inflammatory environment.

Furthermore, C57BL/6 mice have a higher proportion of cross‐presenting CD4^−^/CD8^+^ DCs, which express significantly higher levels of TLR3 than other DC subsets.^53^ TLR3‐deficient mice exhibit exacerbated acute myocarditis after CVB3 infection, highlighting the importance of TLR3 signaling in mounting an effective immune response.^12^

DCs present antigens to lymphocytes, particularly CD4^+^ and CD8^+^ T cells, to form specific immune responses. TLR3^+^ DCs promote T‐lymphocyte proliferation, and in the absence of TLR3, the T‐lymphocyte response tends to shift toward a Th17 profile, which is detrimental to the host.^12^ In subclinical VMC, CD103^+^ DCs prevent the development of overt heart failure by mediating the expansion of antigen‐specific CD8^+^ T cells.^54^ These findings underscore that DCs are not merely “antigen carriers” but strategic regulators that integrate pattern recognition receptor signals and dynamically modulate the differentiation of immune cells.

## NK CELLS

NK cells, a major subset of the innate lymphoid cell family, play a crucial role in the development of CVB3 myocarditis. A mice study has shown that NK cells can limit viral replication by directly killing virus‐infected cardiomyocytes, thereby reducing cardiac damage.^11^ Additionally, NK cells participate in the early inflammatory response, with CXCL10‐mediated recruitment of NK cells to the heart contributing to the suppression of viral replication and reduction of cardiac injury.^11^ In a mice model, the presence of the natural killer group 2, member D (NKG2D) receptor on NK cells has been linked to enhanced antiviral activity against CVB3, associated with better cardiac function.^55^

However, the functionality of NK cells can be influenced by other factors. For instance, in a study using CVB3‐infected A.BY/SnJ mice, an increase in myeloid‐derived suppressor cells was observed in both the spleen and heart compared with uninfected controls.^56^ In vitro experiments confirmed that myeloid‐derived suppressor cells can inhibit the cytotoxic function of NK cells. Specific depletion of myeloid‐derived suppressor cells via anti‐Ly6G antibodies led to a reduction in viral load and cardiac damage, suggesting that myeloid‐derived suppressor cells promote chronic CVB3 myocarditis by impairing NK cell function.^56^ Furthermore, the absence of the transcription factor forkhead box O3a results in increased NK cell activity and enhanced early viral clearance, indicating that forkhead box O3a may negatively regulate NK cell function in CVB3 myocarditis.^57^

As mentioned, although NK cells contribute to the protective effect against viral replication, their phenotype and function can be altered by the microenvironment under viral stimulation, leading to complex regulatory roles in the disease. CVB3 infection significantly induces interferon‐γ expression in NK cells.^58^ Mechanistic studies revealed that estrogen downregulates the expression of T‐bet, a key transcription factor for interferon‐γ production, in CVB3‐stimulated NK cells.^58^

In resistant mice, DCs secrete high levels of IP‐10 and regulated on activation, normal T‐cell expressed and secreted and highly express TLR3, which can enhance the type I interferon response to further promote the recruitment and activation of NK cells; in contrast, impaired antigen‐presenting function and reduced expression of costimulatory molecules of DCs in susceptible mice lead to insufficient activation of NK cells.^53^, ^59^

## LYMPHOCYTES

The cytopathic nature of CVB3 leads to the release of intracellular and surface antigens from the heart, activating T and B lymphocytes and triggering autoimmune responses. During this phase, antigen‐presenting cells present viral or host antigens, activating CD4^+^ T cells to differentiate into Th cell subsets, which then assist B cells in secreting specific antibodies and activate CD8^+^ T cells to differentiate into cytotoxic T lymphocytes that kill virus‐infected cells.

CD4^+^ T cells, activated by antigen peptides and major histocompatibility complex II molecules, primarily differentiate into 4 subtypes: Th1, Th2, Th17, and regulatory T cell (Treg). In CVB3‐induced myocarditis, the proportion of Th1 cells increases. Th1 cells secrete proinflammatory factors, like interferon‐γ, promoting myocardial inflammation. Recent studies using a mice model have demonstrated that the differentiation and function of Th1 cells are highly dependent on the upregulation of glycolysis, and their metabolic profile sustains the proinflammatory phenotype. Conversely, Th2 cells produce anti‐inflammatory factors, such as interleukin‐4, and may have a protective role in reducing CVB3‐induced myocardial damage.^60^ Thus, promoting a Th2 shift could be a therapeutic strategy, although long‐term effects need careful evaluation.

Th17 cells are considered a key driver in CVB3‐related myocarditis. These cells produce proinflammatory factors, such as interleukin‐17, exacerbating the inflammatory process. A study of patient cells has shown that CVB3 transfection of purified CD4^+^ T cells increases the proportion of Th17 cells, interleukin‐17 secretion, and the synthesis of the specific transcription factor retinoic acid‑related orphan receptor γt (RORγT).^61^

In CVB3‐induced myocarditis mice models, a decrease in Treg cell numbers can lead to enhanced autoimmune reactions and worsened disease.^62^ Conversely, exogenous Treg supplementation has been shown to effectively reduce proinflammatory cytokine levels, decrease fibrosis, and improve overall cardiac function.^63^ Recent studies using mice models have demonstrated that CVB3 infection upregulates the long noncoding RNA X‐inactive specific transcript (XIST), which promotes caspase‐1 expression by inhibiting miR‐195‐5p, thereby triggering Treg cell pyroptosis, reducing the Treg/Th17 ratio, and exacerbating inflammation.^64^ Knockdown of XIST can inhibit Treg cell pyroptosis and alleviate myocarditis, providing an epigenetic intervention strategy for restoring Treg cell homeostasis.^64^

In addition to T‐cell subsets, B cells also play a role in the pathogenesis of CVB3 myocarditis. During acute VMC, the number of B cells producing TNF‐α, interleukin‐6, and interleukin‐17 increases, whereas the proportion of interleukin‐4–producing B cells decreases. B‐cell knockout experiments have shown that B‐cell knockout can reduce cardiac damage after CVB3 infection, possibly by inhibiting Th1 and Th17 cell differentiation and promoting Th2 cell differentiation.^60^ B cells can also convert CD4^+^CD25^−^ T cells into Foxp3^+^ and Foxp3‐ regulatory T‐cell subsets. Figure 5B illustrates the role of lymphocytes in the myocardium following CVB3 infection.

## MYOCARDIAL MICROENVIRONMENT AS A THERAPEUTIC TARGET FOR CVB3‐INDUCED MYOCARDITIS

Given the close relationship between CVB3 and the host myocardial microenvironment, where the virus exploits the microenvironment's homeostasis to facilitate its replication, spread, and disease progression, targeting this process may open new avenues for antiviral treatment.

Lovastatin has been found to inhibit CVB3 replication in human ECs,^65^ suggesting that drug interventions may improve outcomes by modulating specific signaling pathways. Beyond directly targeting the virus or its pathologic processes, an increasing number of studies are focusing on how to achieve therapeutic goals by adjusting the activity of immune cells. For example, formoterol can reduce the proinflammatory phenotype produced by monocyte‐derived macrophages by enhancing glutaminase activity,^66^ whereas progranulin can mitigate CVB3‐induced myocarditis by downregulating Th1 and Th17 cells.^67^ These findings indicate that regulating the internal balance of the immune system can also be an effective strategy against CVB3 myocarditis. Gene intervention is another promising antiviral therapy. Conditional gene knockout in specific cell types has already helped identify numerous potential microenvironment‐specific regulatory targets (such as protease‐activated receptor 1, CAR, calpain 4 [CAPN4], and dipeptidase 2), and the regulation of these targets can help alleviate or exacerbate CVB3 myocarditis.

In the exploration of therapies targeting the myocardial microenvironment, cell therapy and targeted intervention strategies have demonstrated promising clinical translation potential because of their unique mechanisms of action. Human right atrial appendage–derived stromal cells reduce fibrosis, improve cardiac function, and downregulate the expression of cardiac chemokines (eg, CCL2 and CCL7), adhesion molecules, and proinflammatory cytokines (TNF‐α and interleukin‐6) in both acute and chronic CVB3‐induced myocarditis models. This indirectly inhibits the infiltration of Ly6C^+^hi monocytes/macrophages, confirming their potent immunomodulatory capacity.^68^

In the field of extracellular vesicle–mediated targeted therapy, relevant research findings have provided new directions for the treatment of CVB3 myocarditis. Recent studies have revealed that large extracellular vesicles derived from lipopolysaccharide‐pretreated cardiomyocytes are enriched in the phosphatase protein phosphatase 2A catalytic subunit, which can induce the polarization of macrophages toward the anti‐inflammatory M2 phenotype while inhibiting M1 polarization both in vivo and in vitro. In CVB3 infection models, extracellular vesicles derived from lipopolysaccharide‐pretreated cardiomyocytes treatment significantly alleviates cardiac inflammation and improves systolic function, indicating that cardiomyocytes can “educate” macrophages and reshape the immune microenvironment by releasing specific vesicles.^69^ Concurrently, a novel prophylactic vaccine named albumin‐binding domain and DC‐targeting peptide exosome has been developed. Based on traditional CVB3 exosome vaccines, this vaccine incorporates an albumin‐binding domain and a DC‐targeting peptide, enabling dual‐targeted delivery to draining lymph nodes and DCs.^70^ Mice immunized with albumin‐binding domain and DC‐targeting peptide exosome exhibit reduced cardiac injury and improved survival rate following CVB3 infection.

Peptide‐based targeted agents have emerged as an important research direction for CVB3 myocarditis treatment because of their high specificity and excellent safety profile. A study first revealed that the 3C protease encoded by CVB3 stabilizes the expression of the antiviral protein viperin in cardiomyocytes by inhibiting the ubiquitin ligase UBE4A. Unexpectedly, viperin does not inhibit viral replication; instead, it activates the SGK1‐KCNQ1 signaling pathway by degrading signal transducer and activator of transcription 1, leading to cardiac electrophysiological disorders and the induction of acute heart failure. Based on this mechanism, researchers designed an interfering peptide named viperin‐STAT1 interaction peptide 1, which can specifically block viperin‐mediated signal transducer and activator of transcription 1 degradation and significantly prevent CVB3‐induced acute heart failure in mice models, providing the first therapeutic peptide directly targeting the key node of virus‐host interaction for clinical translation.^71^

This section summarizes the potential therapeutic drugs (Table 1) and targets (Table 2) discovered in recent years for alleviating CVB3 myocarditis through the regulation of the myocardial microenvironment.

**Table 1:** Potential Therapeutic Agents for Alleviating CVB3 Myocarditis by Modulating the Myocardial Microenvironment

Target cell	Research model	Agent name	Influence and mechanism	References
Cardiomyocytes	Mouse animal model	VS‐IP1	Specifically blocks viperin‐mediated STAT1 degradation; prevents CVB3‐induced AHF in mice models (viperin activated SGK1‐KCNQ1 signaling pathway by degrading STAT1, leading to cardiac electrophysiological disorders and AHF)	71
Cardiomyocytes	Mouse animal model	BK1.3	Derived from tick salivary protein evasin; blocks multiple proinflammatory chemokines (CCL2, CCL3, CCL7, and CCL8); at 5‐mg/kg dose, reduces myeloid cell infiltration in myocardium and liver during subacute phase, improves liver injury indicators, and alleviates cardiac systolic dysfunction in CVB3 mice models.	72
Fibroblast	Mouse animal model, in vitro model	Calpain inhibitor I (ALLN)	Inhibit the migration of fibroblasts in vitro	73
Endothelial cell	In vitro model	Lovastatin	Lovastatin downregulates the expression of CAR through a Rac1/Cdc42‐dependent mechanism, thereby reducing the replication of CVB3 in HUVECs.	65
Macrophage	Mouse animal model, in vitro model	Ruxolitinib	Ruxolitinib treatment inhibits the activation of the JAK–STAT pathway and suppresses the elevation of mRNA levels of multiple cytokines in BMDMs.	74
	Mouse animal model, in vitro model	Ivermectin	Ivermectin inhibits the nuclear translocation of NF‐κB/p65 in macrophages and downregulates the expression of proinflammatory cytokines.	75
	Mouse animal model, in vitro model	Formoterol	Formoterol enhances glutaminase activity in monocyte‐derived macrophages, thereby reducing the proinflammatory phenotype.	66
	Mouse animal model	RAA‐CardAPs	Reduce fibrosis, improve cardiac function, downregulate the expression of cardiac chemokines (eg, CCL2 and CCL7), adhesion molecules, and proinflammatory cytokines (TNF‐α and IL‐6) in acute and chronic CVB3‐induced myocarditis models; indirectly inhibit the infiltration of Ly6C^+^hi monocytes/macrophages	68
	Mouse animal model, in vitro model	C‐lEVLPS	Enriched in phosphatase PP2AA; induce macrophage polarization toward anti‐inflammatory M2 phenotype and inhibit M1 polarization in vivo and in vitro; alleviate cardiac inflammation and improve systolic function in CVB3 infection models	69
Dendritic cells	Mouse animal model	AD‐Exo	Incorporates ABD and dendritic cell–targeting peptide (DCpep) for dual‐targeted delivery; promotes antigen enrichment and DC activation, recruits NK cells, increases IFN‐γ–secreting immune cells; enhances anti‐CVB3 protective efficacy via DC‐NK cell axis immune activation; reduces cardiac injury and improves survival rate in immunized mice after CVB3 infection	70
Lymphocyte	Mouse animal model, in vitro model	Progranulin	Progranulin regulates the differentiation of Th1 and Th17 cells by inhibiting the JAK–STAT pathway.	67
	Mouse animal model	Fasudil	Fasudil reduces CVB3 replication and downregulates ROCK2/IL‐17 expression, while promoting a shift of T‐cell subsets from Th17 to Treg in cardiac tissue.	76
	Mouse animal model, in vitro model	α7nAChR agonist pnu282987	In VMC mice, the percentage of Th1 and Th17 cells was downregulated, whereas the percentage of Th2 and Treg cells was upregulated on administration of PNU282987.	77
Neutrophils	Mouse animal model	Astragaloside IV (AS‐IV)	Inhibits NETs via regulating IRF7/NLRP3 axis (blocks IRF7‐NLRP3 promoter binding), thereby alleviating CVB3‐induced myocardial inflammation, fibrosis, and injury, and improving cardiac function (a potential TCM monomer targeting neutrophil inflammatory pathway)	78
Neutrophils	Mouse animal model	α‐Lipoic acid	Induces metabolic reprogramming in neutrophils to produce acetyl‐CoA, upregulates Ym‐1 via STAT6 acetylation; Ym‐1^+^ neutrophils polarize macrophages to reparative phenotype. Efficacy depends on neutrophil subsets (anti‐Ly6G depletion reverses effect), supporting neutrophil heterogeneity in tissue repair	79

**Table 2:** Potential Therapeutic Targets for Regulating the Myocardial Microenvironment to Alleviate CVB3 Myocarditis

Target cell	Target factor	Research model	Intervention method	Influence and mechanism	References
Fibroblast	Calpastatin	Mouse animal model, in vitro model	Calpastatin transgenic mice strain (Tg‐CAST)	Overexpression of calpastatin significantly ameliorated myocardial injury induced by CVB3 infection in transgenic mice.	73
	PAR1	Mouse animal model, in vitro model	Mice with specific knockout of PAR1 in cardiac fibroblasts. PAR1ΔCF (PAR1^fl/fl^;TCF21^ETR2Cre^) mice	The heart exhibited increased CVB3 genome, inflammatory infiltration, macrophages, and inflammatory mediators. This promoted autophagy in mice embryonic fibroblasts, which helps to explain the regulation of viral replication by PAR1.	80
	miR‐214	Mouse animal model, in vitro model	miR‐214 agomir and antagomir	miR‐214 bidirectionally inhibits the expression of p53 and PTEN, thereby activating the PI3K/Akt signaling pathway. Downregulation of miR‐214 suppresses the infiltration of inflammatory cells and fibroblasts in the myocardial tissue of VMC mice and promotes fibroblast apoptosis.	33
Endothelial cell	CAR	Mouse animal model, in vitro model	Endothelial cell–specific CAR knockout (CAR‐eKO) mice	The ablation of CAR did not significantly increase CVB3‐induced myocarditis in mice. However, the tissue viral titer in CAR‐eKO mice was significantly elevated.	81
	FKN	In vitro model	FKN shRNA	Silencing of FKN inhibits CVB3 replication in endothelial cells.	82
Macrophage	CAPN4	Mouse animal model, in vitro model	CAPN4 conditional knockout mice (CAPN4^flox/flox^; LYZ2‐Cre, CAPN4‐cKO)	CAPN4 increases M1‐type macrophage polarization and inhibits M2‐type macrophage polarization via the CHOP‐STAT1/STAT3 signaling pathway, thereby mediating CVB3‐induced myocardial inflammation and injury.	83
	Dpep2	Mouse animal model, in vitro model	Macrophage‐specific Dpep2 knockout mice (Dpep2^fl/fl^LyzM‐Cre^+^)	Dpep2‐deficient BMDMs produce more inflammatory cytokines after CVB3 stimulation, which is dependent on their regulation of the NF‐κB signaling pathway.	84
	SPP1	Mouse animal model	SPP1 inhibitor (SEI)	Spp1^+^ macrophages can self‐recruit, and SPP1 derived from macrophages exacerbates myocardial immune injury by promoting the high expression of Ccl2/Ccl7 in fibroblasts.	43
	Lnc AK083884	Mouse animal model, in vitro model	si‐AK083884	AK083884 derived from M2 exosomes promotes M2 macrophage polarization and protects mice from CVB3‐induced VM.	85
Neutrophil	IL‐37	Mouse animal model, in vitro model	AAV9‐IL‐37	IL‐37 can alleviate acute VMC symptoms caused by CVB3, reduce inflammatory cell infiltration, improve cardiac function, and inhibit the formation of NETs in the myocardium.	51
Natural killer cells	Foxo3	Mouse animal model, in vitro model, Human research model	Foxo3knockout mice	The absence of FOXO3a leads to increased NK cell activity and enhanced early viral clearance.	57
Dendritic cells	TLR3	Mouse animal model, in vitro model	Adoptive transfer of TLR3^+^ dendritic cells	The absence of TLR3 leads to a skewing of T‐lymphocyte responses toward the Th17 phenotype; adoptive transfer of TLR3^+^ dendritic cells to TLR‐deficient mice can slightly improve their survival rate after CVB3 infection.	12
Lymphocyte	IL‐27	Mouse animal model	Recombinant mice IL‐27	IL‐27 inhibited the frequency of Th17 cells and the production of IL‐17, as well as Th17‐related proinflammatory cytokines in cardiac tissue	86
	CD80	Mouse animal model, in vitro model	CD80 mAb	CD80 mAb treatment significantly inhibited the differentiation of Th17 cells and the expression of ROR‐γt mRNA	87

DPEP2, Dipeptidase 2

Specifically, Table 1 systematically summarizes potential therapeutic agents that can ameliorate CVB3‐induced myocarditis by regulating the myocardial microenvironment. Categorized by their target cell types, cardiomyocytes,^71^, ^72^ cardiac fibroblasts,^73^ ECs,^65^ macrophages,^66^, ^68^, ^69^, ^74^, ^75^ DCs,^70^ lymphocytes,^67^, ^76^, ^77^ and neutrophils,^78^, ^79^ this table clearly presents the research progress of different agents in targeting various cellular components of the myocardial microenvironment and intervening in inflammatory responses and myocardial injury. Table 2 systematically delineates the key potential therapeutic targets for alleviating CVB3‐induced myocarditis by regulating the myocardial microenvironment. It comprehensively summarizes candidate targets and research evidence for precision intervention in myocarditis, which are present on key cell types, including cardiac fibroblasts,^33^, ^73^, ^80^ ECs,^81^, ^82^ macrophages,^43^, ^83^, ^84^, ^85^ neutrophils,^51^ NK cells,^57^ DCs,^12^ and lymphocytes.^86^, ^87^

## STAGE‐DEPENDENT PATHOGENESIS AND PRECISION TARGETING IN CVB3‐INDUCED MYOCARDITIS

In CVB3‐induced myocarditis, the pathologic mechanisms, key cellular components, and signaling pathways differ drastically across distinct stages, thus necessitating highly stage‐specific intervention strategies to maximize clinical efficacy.

In the early infection phase (0–3 days), the core objectives are to restrain viral replication and protect cardiomyocytes, while avoiding excessive suppression of the innate immune response. Priority should be given to targeting pathways involved in viral entry and replication, including the CAR/decay‐accelerating factor receptors, retinoic acid–inducible gene I/TLR3, and cyclic GMP‐AMP synthase–stimulator of interferon genes pathways, which mediate the early antiviral interferon response. Studies have demonstrated that TRIM21 facilitates type I interferon production by enhancing MAVS‐mediated interferon regulatory factor 3 activation, thereby effectively limiting viral replication^88^; in addition, small‐molecule inhibitors targeting viral proteins, such as the 2A protease or RNA helicase 2C, have been proven to markedly reduce viral load and alleviate acute myocardial injury.^89^, ^90^ Notably, broad anti‐inflammatory interventions are not recommended at this stage, as innate immune components, such as NK cells, are indispensable for early viral clearance; early administration of interleukin‐2 can enhance NK cell activity, reduce viral titer, and improve survival rates.^91^

On entering the acute inflammation phase (3–14 days), especially after the viral replication peak (3–5 days), the immune response shifts from antiviral dominance to inflammatory amplification, where indiscriminate anti‐inflammatory treatment may impede viral clearance. The key cellular players in this phase include monocytes/macrophages, CD8^+^ T cells, and neutrophils, with core pathways encompassing NF‐κB, CCL2/C‐C chemokine receptor 2, and the interleukin‐21 receptor/Tfh/B axis. The focus shifts to targeted immunomodulation rather than global inflammation suppression: for instance, blocking interleukin‐21 receptor signaling reduces Tfh cell expansion and autoantibody production, thereby mitigating myocardial damage without compromising viral clearance.^1^ Recent studies have also revealed that cardiac fibroblasts are activated into “inflammatory fibroblasts” by interleukin‐1β as early as day 3 after infection, driving myeloid cell infiltration, suggesting that early targeting of interleukin‐1 signaling in fibroblasts may limit subsequent inflammatory cascades. Furthermore, β‐arrestins modulate the cyclic GMP‐AMP synthase–stimulator of interferon genes pathway in cardiomyocytes to regulate systemic immune cell recruitment, representing another potential therapeutic target.^5^

The chronic repair/fibrosis phase (>14 days) is characterized by viral clearance, with pathologic focus shifting to myocardial fibrosis and remodeling, during which profibrotic pathways, including TGF‐β1/Smad, SPP1/CCL2, and endothelial‐to‐mesenchymal transition, predominate. Targeting fibroblast activation and extracellular matrix deposition becomes the central strategy. Evidence confirms that even late administration of interleukin‐1β blockade significantly reduces collagen deposition and the expression of fibrosis‐related genes (eg, Tgf‐β and Timp‐1 [tissue inhibitor of metalloproteinase]) and reverses adverse myocardial remodeling.^1^ Other effective targets include the following: inhibiting SPP1 to downregulate the matrix metalloproteinase‐3/TIMP1/urokinase‐type plasminogen activator (uPA)axis and alleviate fibrosis^43^; activating AMP‐activated protein kinase to suppress CVB3‐induced fibroblast proliferation and collagen secretion^92^; and regulating the miR‐214/p53/phosphatase and tensin homolog axis to inhibit endothelial‐mesenchymal transition and fibrogenesis.^33^ Notably, cardiac dysfunction may persist in some models despite resolved inflammation and attenuated fibrosis, indicating that comprehensive repair mechanisms, including metabolic remodeling and contractile function recovery, should be considered alongside antifibrotic interventions.

**Figure 6:**
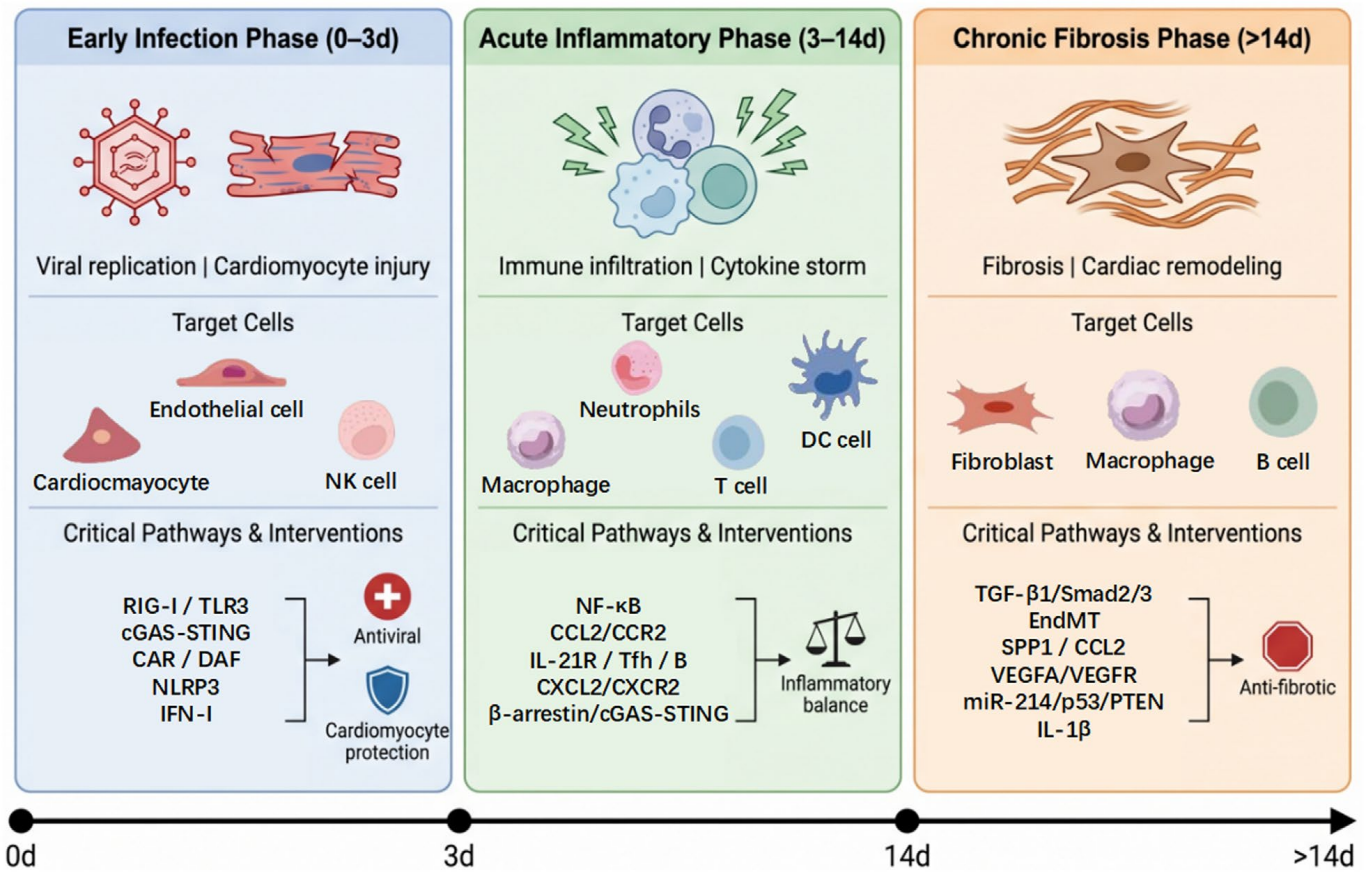
Stage‐specific pathogenesis and targeted interventions in CVB3‐induced myocarditis. This schematic timeline illustrates 3 consecutive pathologic phases of CVB3‐induced myocarditis, summarizing stage‐specific core features, preferential target cells, key signaling pathways, and therapeutic principles. The early infection phase (0–3 days) is marked by viral replication and cardiomyocyte injury, prioritizing antiviral and cardioprotective strategies via RIG‐I/TLR3, cGAS‐STING, and IFN‐I pathways. The acute inflammatory phase (3–14 days) features immune infiltration and cytokine storm, requiring targeted immunomodulation via NF‐κB, CCL2/CCR2, and β‐arrestin/cGAS‐STING pathways. The chronic fibrosis phase (>14 days) presents progressive fibrosis and remodeling, requiring antifibrotic interventions via TGF‐β1/Smad2/3 and EndMT pathways. CAR indicates coxsackievirus and adenovirus receptor; CCL, chemokine (C‐C motif) ligand; CCR, C‐C chemokine receptor 2; cGAS, cyclic GMP‐AMP synthase; CXCL, chemokine (C‐X‐C motif) ligand; CXCR, chemokine (C‐X‐C motif) receptor; DAF, decay‐accelerating factor; DC, dendritic cell; EndMT, endothelial‐to‐mesenchymal transition; IFN, interferon; IL‐21R, interleukin‐21 receptor; NF‐κB, nuclear factor‐κB; NK, natural killer; NLRP3, NOD‐like receptor family pyrin domain containing 3; PTEN, phosphatase and tensin homolog; RIG‐I, retinoic acid–inducible gene I; SPP1, secreted phosphoprotein 1; STING, stimulator of interferon genes; TGF‐β, transforming growth factor‐β; TLR, toll‐like receptor; VEGFA, vascular endothelial growth factor A; and VEGFR, vascular endothelial growth factor receptor.

In summary, precise stage‐specific targeting, antiviral therapy in the early phase, immunomodulation in the intermediate phase, and antifibrotic therapy in the late phase, is the pivotal strategy to improve the clinical outcomes of CVB3‐induced myocarditis. This stage‐dependent therapeutic framework is schematically illustrated in Figure 6, providing a clear visual guide for prioritizing precision interventions based on disease progression.

## CLINICAL TRANSLATION CHALLENGES OF MYOCARDIAL MICROENVIRONMENT‐TARGETED THERAPY

Although targeting the myocardial microenvironment has shown encouraging preclinical results in the treatment of CVB3‐induced myocarditis, its translation from the laboratory to the clinic still faces multiple practical obstacles.

First, the selection of intervention timing constitutes a core challenge. The course of viral myocarditis is clearly time dependent: the acute phase is dominated by direct viral replication and cell lysis, followed by the gradual predominance of immune‐mediated inflammatory responses. During the active viral replication phase (typically 3–7 days after infection), premature administration of immunomodulatory or inhibitory interventions may alleviate inflammatory injury but simultaneously compromise the host's innate immune response, delay viral clearance, and consequently exacerbate myocardial damage. For instance, the NLRP3 inflammasome not only participates in the maturation and release of proinflammatory cytokines but also indirectly restricts viral spread under certain conditions by recruiting immune cells and promoting an antiviral state; the glycolytic pathway provides the energetic basis for immune cell activation and proliferation, and its blockade may impair effector cell function; whereas TGF‐β may also be involved in regulating the balance between immune homeostasis and tissue repair in the early stage. Studies have demonstrated that certain immunomodulators, such as histone deacetylase inhibitors, unexpectedly promote autophagosome formation in CVB3 infection models, thereby enhancing viral replication and aggravating myocardial apoptosis and inflammation.^93^

Second, safety and off‐target effects represent unavoidable bottlenecks in clinical translation. Most microenvironment‐targeting strategies, whether small‐molecule drugs, gene editing, cell therapy, or engineered exosomes, lack organ specificity. For example, drugs designed to regulate macrophage polarization or T‐cell differentiation (eg, formoterol or progranulin analogs) may systemically disrupt systemic immune homeostasis, increasing the risk of secondary infection or autoimmune disorders. More complexly, the myocardial microenvironment is composed of multiple cell types with highly dynamic spatial heterogeneity and signal crosstalk. Interventions targeting a specific cell subset or signaling pathway may indirectly affect other cell types via paracrine signaling or metabolic remodeling, leading to unpredictable off‐target effects. For example, strategies aimed at inhibiting M1‐type macrophages may simultaneously compromise their antiviral capacity, whereas excessive promotion of M2‐type polarization may accelerate the fibrotic process.

Although recent studies have attempted to initiate immunomodulation in the late stage of infection to avoid the peak of viral replication, the precise definition of the “therapeutic window” for individual patients still lacks reliable biomarker support. In addition, most preclinical models use inbred mice and standardized viral strains, which cannot fully mimic the genetic diversity, comorbidities, and mixed infection backgrounds of the human population. As a result, the significant therapeutic effects observed in animal experiments are often attenuated or negated in real‐world settings.

## THE PROSPECTS OF CVB3 THERAPY AND DIAGNOSIS MEDIATED BY CARDIAC MICROENVIRONMENT

In recent years, the development of CVB3 vaccines targeting the myocardial microenvironment has evolved from conventional antigen delivery toward precision immune modulation. As mentioned earlier, there is the dual‐target exosome vaccine albumin‐binding domain and DC‐targeting peptide exosome. Intelligent delivery systems capable of precisely modulating the local immune microenvironment can transcend the limitations of traditional vaccines for target specificity, immune response intensity, and safety.

Notably, the in‐depth exploration of the myocardial immune microenvironment has been greatly facilitated by technological advancements in single‐cell and spatial omics, which have uncovered the spatial heterogeneity of the immune microenvironment and the landscape of cell–cell interactions. A 2021 study based on a mouse model^26^ revealed, through single‐cell transcriptome sequencing (single‐cell RNA sequencing), the first animal‐level characterization of immune remodeling in viral myocarditis. The study compared the cellular profiles of myocardial tissues from healthy and CVB3‐infected mice, demonstrating a significant increase in myeloid cells (particularly M2 macrophages), CD4^+^/CD8^+^ T cells, and fibroblasts, alongside a reduction in NK cells, innate lymphoid cells, and B cells. These findings sufficiently elucidate the dynamic changes in cell types and functional states that traditional histology cannot capture. A study for the first time accomplished 3‐dimensional reconstruction of the entire heart in mice with acute CVB3 myocarditis through the CO‐registration of spatial data with anatomy workflow, and identified that T‐cell– and macrophage‐dominated immune foci are distributed in distinct anatomic regions within the 3‐dimensional space, suggesting the existence of functional zonation in the local immune microenvironment.^94^ A concurrent spatiotemporal transcriptomic study indicated that in a fulminant myocarditis model, epicardial mesothelial cells serve as the primary early infection targets of CVB3, which subsequently activate macrophages and recruit proinflammatory Inflammatory_Mac and T cells; among these, CD8^+^ effector T cells upregulate Spi1 expression by secreting interferon‐γ, thereby exacerbating cardiomyocyte death, whereas intravenous immunoglobulin can effectively inhibit this axis and improve prognosis.^9^ The synergistic advantage of spatial omics and single‐cell technologies lies in anchoring molecular information to 3‐dimensional anatomic structures. Single‐cell sequencing excels at unbiased capture of rare cell types and their transcriptional states, whereas spatial omics preserves the in situ localization and neighboring interactions of cells within the tissue microenvironment. The combination of these 2 approaches not only overcomes the limitation of traditional pathologic sections lacking molecular depth but also compensates for the spatial dimensionality deficiency in single‐cell data loss.

In‐depth analysis and precise regulation of the myocardial microenvironment constitute the core of research on CVB3‐induced myocarditis, and innovations in in vitro models provide crucial support for this research. Cardiac organoids, with their high capacity to simulate the myocardial microenvironment, have emerged as a vital bridge connecting basic research and clinical application. Cardiac organoids overcome the dual limitations of traditional 2‐dimensional cell culture and animal models by reconstructing in 3‐dimensional space the multicellular interaction network and dynamic pathologic processes of human myocardial tissue. This enables in situ observation of viral transmission pathways between different cell types, establishment of local inflammatory factor gradients, and spatial heterogeneity of matrix remodeling following CVB3 infection. More importantly, by integrating patient‐derived induced pluripotent stem cells, organoids can capture the influence of individual genetic background on viral susceptibility and fibrotic propensity, providing a controllable experimental system for elucidating clinical heterogeneity. A recent study constructed 3‐dimensional engineered heart tissue composed of induced pluripotent stem cell–derived cardiomyocytes and fibroblasts. Under profibrotic stimulation, this engineered heart tissue successfully simulated the decrease in contractile function and increase in passive tension, providing a standardized platform for investigating fibrotic mechanisms and high‐throughput screening of antifibrotic compounds.^95^

Meanwhile, the integration of artificial intelligence (AI) technology has significantly enhanced the analytical capability of this system. Latest research indicates that AI algorithms can quantitatively assess the inflammatory level in organoids and optimize therapeutic responses in combination with metabolic intervention strategies, achieving a closed‐loop research from phenotypic identification to mechanistic intervention. Additionally, this platform supports high‐throughput screening of antiviral drugs, immunomodulators, and antifibrotic compounds, while simultaneously evaluating their potential toxicity to myocardial function and microenvironmental homeostasis, thereby providing data support for the formulation of individualized treatment regimens. Despite remaining challenges, such as standardization of maturity and immune compatibility, the AI‐integrated cardiac organoid system is gradually becoming a core tool for dissecting CVB3‐host interactions and developing microenvironment‐targeted therapies, propelling cardiovascular disease research toward precision medicine.

The integration of AI with cutting‐edge biotechnology is reshaping the research landscape of CVB3‐induced myocarditis. Confronted with massive multidimensional data generated by advanced technologies, such as organoids, single‐cell sequencing, and spatial transcriptomics, AI has emerged as a core tool for deciphering the complexity of the myocardial microenvironment in CVB3 viral myocarditis. By integrating single‐cell transcriptomic, proteomic, and metabolomic data, AI algorithms can unbiasedly identify newly emerged cell subpopulations or transitional states during infection (for instance, predicting previously undiscovered ligand‐receptor pairs between fibroblasts and macrophages), thereby pinpointing the core signaling hubs that drive fibrosis.^26^ For example, in studies of immune‐related myocarditis, researchers have used single‐cell RNA sequencing combined with AI‐driven cell–cell communication analysis tools (eg, CellChat) to identify a critical inflammatory axis composed of CXCL9^+^ fibroblasts and atypical chemokine receptor 1^+^ ECs. These cell subsets reshape the local immune infiltration profile by secreting chemokines and have been verified to exhibit pathologic specificity in both viral and autoimmune myocarditis models.^96^

Another key capability of AI is reflected in the integration of multi‐omics data. A recent study integrating transcriptomics and metabolomics analyzed CVB3‐infected mouse models using AI algorithms, which not only identified hub genes associated with mitochondrial dysfunction, such as mitogen‐activated protein kinase 8 and nicotinamide phosphoribosyltransferase (NAMPT), but also validated the reprogramming characteristics of central carbon metabolism pathways via targeted metabolomics.^97^

For spatial transcriptomics, AI algorithms have been applied to dissect the spatial heterogeneity of cell distribution and gene expression within inflammatory foci. For instance, a 2025 study using the CO‐registration of spatial data with anatomy 3‐dimensional reconstruction pipeline coupled with spatial transcriptomics demonstrated that, through machine learning–driven spatial clustering and regional annotation, researchers successfully identified a spatial pattern in the left ventricular wall of CVB3‐infected mouse hearts, where T‐cell–enriched regions and macrophage‐dominant zones were segregated anteriorly and posteriorly. It was further revealed that anterior T‐cell hotspots specifically highly expressed collagen type I and T‐cell chemokines, suggesting a functionally compartmentalized immune–stromal interaction network in the local microenvironment.^98^

In the broader field of myocardial diseases, AI has been used to guide the prediction of organoid differentiation trajectories, cell fate simulation, and drug response modeling, capabilities that can be seamlessly transferred to CVB3 infection scenarios.

## CONCLUDING REMARKS AND OUTLOOK

CVB3 myocarditis is a significant threat to human health, with its pathogenesis closely linked to the reprogramming of the myocardial microenvironment. Although current research has confirmed the central role of immune imbalance, inflammation, and fibrosis in disease progression, specific therapeutic strategies targeting the dynamic regulation of the microenvironment remain scarce. This review systematically analyzes the interactions among ECs, fibroblasts, and various immune cells within the myocardial microenvironment, revealing how viral invasion triggers cellular phenotypic changes (such as proinflammatory macrophage polarization and abnormal fibroblast activation) that disrupt cell communication, induce metabolic disorders, and promote paracrine abnormalities, collectively driving myocardial injury and fibrosis. Future research should focus on the following: elucidating the molecular logic of the spatiotemporal dynamics of microenvironmental components, especially the characteristics of cell interactions in viral replication hotspots; clarifying the positive feedback mechanisms between microenvironmental homeostasis and immune responses, and unraveling how mechanobiological signals exacerbate pathologic remodeling; and developing intelligent drug delivery systems targeting specific cell subpopulations (such as M2 macrophages and fibroblasts), while also considering the bidirectional regulatory effects of therapeutic interventions on microenvironmental homeostasis. This will provide theoretical support for overcoming the current treatment dilemma, which primarily focuses on symptom management.

## Disclosures

None.
